# Prevalence and Mortality of Life-Threatening and Life-Shortening Diseases in Children and Adolescents in Germany

**DOI:** 10.1177/00099228241264123

**Published:** 2024-07-23

**Authors:** Nadja Melina Burgio, Sven Jennessen

**Affiliations:** 1 Department of Rehabilitation Sciences, Faculty of Humanities and Social Sciences, Humboldt-Universität zu Berlin, Berlin, Germany

**Keywords:** pediatrics, life-limiting illness, life-threatening, diagnosis code, cause-of-death statistics, regional differences, palliative medicine

## Abstract

This study provides prevalence and mortality data for 0- to 19-year-old children and adolescents with medically documented life-threatening and life-shortening diagnoses in Germany. A secondary data analysis of more than 12 million insured persons documented by the statutory health insurance funds in Germany from 2014 to 2019 was conducted in collaboration with the German Association of Statutory Health Insurance Funds (GKV-SV) and the Institute for Applied Health Research Berlin (InGef), whose data sets vary in collection methods. Diagnosis prevalence and mortality were calculated based on selected International Classification of Diseases, 10th Revision (ICD-10) codes reported in inpatient and outpatient care settings. In Germany, the diagnosis prevalence of life-threatening and life-shortening diseases in children and adolescents ranges between 319 948 (InGef—adapted Fraser list) and 402 058 (GKV-SV). These diagnoses can be differentiated into different disease groups (Together-for-Short-Lives [TfSL] 1-4). The TfSL-1 group in which curative treatment can be feasible represents the largest one, with 190 865 persons. In 2019, approximately 1458 children and adolescents with life-threatening and life-shortening diseases died. The current diagnostic and mortality data of affected children and adolescents in Germany serve as the essential foundation for further research into the health care of the target group.

In England, in 2012, Fraser and colleagues conducted a prevalence study on life-threatening and life-shortening diseases among children and adolescents aged 0 to 19 years, covering the period from 2000/2001 to 2009/2010.^1^ As there were no data available for these disease groups in Germany, the English data were extrapolated and applied to Germany, assuming similar epidemiology between the 2 countries.^2^ In this extrapolation, it was previously assumed that, among a total population of 15.6 million children and adolescents aged 0 to 19 years in Germany, approximately 50 000 individuals live with life-shortening or life-threatening illnesses,^2^ a figure communicated by relevant actors in the German health care system.^3,4^

The PraeKids project, in collaboration with the German National Association of Health Insurance Funds (GKV-SV), the Institute for Applied Health Research Berlin GmbH (InGef), and pediatric palliative physicians, collected data from 2014 to 2019 on the number of affected children and adolescents aged 0 to 19 years in Germany for the first time.^5^ Furthermore, the mortality rate of the patients and the most frequent diagnoses of the deceased patients within the respective year of analysis were recorded using InGef data. In summary, the aim of the study is to assess the number of children and adolescents medically diagnosed with life-threatening and life-shortening conditions in Germany, as well as the mortality rates among them. The focus in the following is on data from 2019 as it represents the most up-to-date information available and therefore serves as a basis for future research in the area of health care.

As the English studies included patients up to the age of 19 years and these data form the basis for the prevalence values of children and adolescents used in the German palliative care landscape,^1,2^ this study also focused on this age range with the aim of international data comparability.

## Methods

Following the study by Fraser et al,^1^ the International Classification of Diseases, 10th Revision (ICD-10) codes served as the basis for determining diagnosis prevalence and mortality of life-threatening and life-limiting diseases in this German study. First, the diagnosis list from England was examined with palliative care physicians to determine the extent to which the ICD-10 codes could be classified as life-threatening and life-shortening and could be assigned to one of the 4 Together-for-Short-Lives (TfSL) groups:

TfSL-1: Life-threatening diseases with the possibility of curative therapy, which may fail (eg, tumor diseases).
TfSL-2: Diseases that cannot be cured but have life-prolonging treatment options (eg, cystic fibrosis).
TfSL-3: Progressive diseases without life-prolonging therapy but with palliative care options (eg, adrenoleukodystrophy).
TfSL-4: Irreversible, nonprogressive diseases, mainly neurological, leading to premature death (eg, hypoxic encephalopathy).^6,7^

Four participating physicians reviewed the ICD-10 codes and discussed any codes that were unclear in terms of their classification. The resulting “adapted Fraser list” excluded codes that the physicians deemed should not be included in the prevalence calculation, eg, H11.1 Conjunctival degenerations and deposits and Q77. Achondroplasia. In addition, a new review of the ICD-10 codes was conducted to consider updates and corrections. The resulting list included codes from Fraser et al^1^ as well as newly added codes (This new list can be found in the research report on the PraekKids project at https://edoc.hu-berlin.de/bitstream/handle/18452/25451/PraeKids_Abschlussbericht_2022.pdf.).

Data from 109 statutory health insurance funds (GKV-SV)^8^ and InGef’s database, representing 8.8 million insured individuals,^9^ were collected and analyzed. The new list by Burgio and Jennessen served as the basis for both the prevalence and mortality surveys.

## German Association of Statutory Health Insurance Funds Data for the Prevalence Survey

According to its own information, the staff unit contract analysis of GKV-SV data uses extracted master data for insured persons and collective contract billing data for statutorily insured persons to determine the diagnosis prevalence. Data for this sample are specifically collected by the GKV-SV for each data year.

For the calculation of the GKV-SV prevalence figures, each child or adolescent diagnosed with one of the conditions on the new list was counted once, even though other life-threatening or life-shortening diagnoses were made. The prevalence values of the GKV-SV were extrapolated to the population by multiplying the percentage of GKV-insured persons with at least one of the diseases by the number of the individuals aged 0 to 19 years^10^ without further weighting by region or age and sex groups.^5^

The inpatient diagnosis prevalence of the GKV-SV cannot be depicted as only diagnosis cases and not the insured persons in the care area can be counted separately. No prevalence data can be derived from this.

## Institute for Applied Health Research Berlin Data for the Prevalence and Mortality Survey

The prevalence and mortality survey by InGef was conducted for each individual year during 2014 to 2019 through a cross-sectional analysis. The following inclusion criteria were applied:

Patients who were insured from January 01 or from birth to December 31 or until death within the respective year of analysis;
Patients with a maximum age of 19 years on December 31 of the respective year of analysis.

With regard to the case definition, patients with at least 1 main or secondary inpatient diagnosis and/or 2 confirmed outpatient diagnoses in different quarters of the respective year of analysis (M2Q criterion) were considered. The prevalence of diseases in TfSL groups 1 to 4 and the adapted Fraser list were also surveyed. The prevalence values of InGef were extrapolated to the population aged 0 to 19 years^10^ without further weighting by region or age and sex groups.^5^

In an additional analysis, the mortality of children with life-threatening and life-shortening diseases was also recorded based on the case definitions for the ICD-10 list by Burgio and Jennessen.

On the basis of the determined mortality rate, the most frequent diagnoses made in deceased patients within the respective year of analysis could be recorded. On one hand, the proportions of deceased patients who received at least 1 diagnosis from each diagnosis category, such as respiratory or hematological diseases, were determined; on the other hand, all individual diagnoses that were used for all case definitions were also taken into account.

## Results

**Figure 1.**
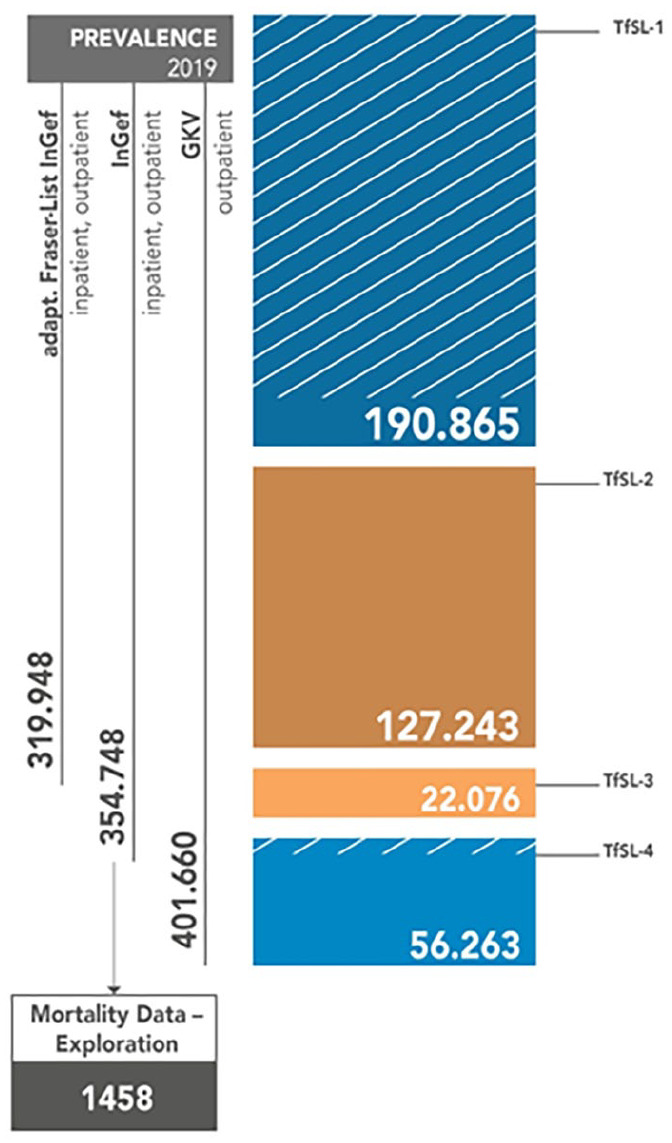
Prevalence range and mortality rate of life-threatening and life-shortening diseases in children and adolescents in Germany in 2019. The figure shows hatched lines for a proportion of diagnosis in TfSL-1 and TfSL-4 that cannot be a clearly quantified result in a shortening of life. Source: Compiled by the authors. Abbreviation: GKV, German Association of Statutory Health Insurance Funds; InGef, Institute for Applied Health Research Berlin GmbH.

In Germany, the prevalence range of medically documented life-threatening and life-shortening diseases in children and adolescents in 2019 was between 319 948 (InGef—adapted Fraser list) and 402 058 (GKV-SV, outpatient), considering TfSL groups 1 to 4^5^ (Figure 1).

The mortality values will be presented in more detail in the following. It can be noted that a total of 198 life-shortening and life-threatening diagnoses were made in 111 patients across the four TfSL groups (Table 1). The largest proportion was found in TfSL-1 (88.29%), followed by TfSL-2 (36.04%), TfSL-4 (19.82%), and finally TfSL-3 (34.23%; Table 1). It is significant to acknowledge that the TfSL groups 1 to 4 consist of various diseases; hence, there may be multiple diagnoses for the same patients. The secondary data analysis of InGef showed also that respiratory insufficiency was the most frequent diagnosis. This was followed by malignant neoplasms and cardiac insufficiency. Furthermore, Table 2 of the InGef data demonstrates that there were 411 diagnoses of life-threatening and life-shortening diseases per 100 000 in 2019. Multiplying the quotient of both values by the current prevalence value of 354.748 from InGef, one obtains a mortality rate of 1458 children and adolescents in that year (Tables 2 and 3; Figure 1).

**Table 1.** Mortality Data—Diagnostic Groups (InGef-Data, 2019).

2019				
	ICD-10-GM	Label/list	n	%
	Total deceased patients		111	100.00
List of diseases	Fraser list		108	97.30
	Neurological		40	36.04
	Hematological		18	16.22
	Oncological		32	28.83
	Metabolic		8	7.21
	Respiratory		55	49.55
	Cardiovascular		41	36.94
	Gastrointestinal		6	5.41
	Urogenital		23	20.72
	Perinatal		25	22.52
	Congenital		16	14.41
	Others		<5	—
	TfSL-1		98	88.29
	TfSL-2		40	36.04
	TfSL-3		22	19.82
	TfSL-4		38	34.23

Abbreviations: ICD-10, International Classification of Diseases, 10th Revision; InGef, Institute for Applied Health Research Berlin GmbH.

**Table 2.** Mortality Data (InGef-Data, 2014 to 2019).

Year	Total patients	Deceased	Percentage of deceased in %	Per 100 000	95% CI lower limit	95% CI upper limit
2014	24.562	98	0.40	399	324	486
2015	25.288	108	0.43	427	350	516
2016	25.984	100	0.38	385	313	468
2017	26.628	92	0.35	346	279	424
2018	27.244	104	0.38	382	312	463
2019	27.034	111	0.41	411	338	494

Abbreviation: InGef, Institute for Applied Health Research Berlin GmbH.

**Table 3.** Prevalence and Mortality Data (InGef -Data, 2014 to 2019).

Year	Prevalence rate per 100 000	Prevalence—InGef (extrapolated)	Mortality
2014	2.103	310.266	1.238
2015	2.152	324.330	1.385
2016	2.241	341.112	1.313
2017	2.265	345.465	1.196
2018	2.300	351.769	1.344
2019	2.314	354.748	1.458

Abbreviation: InGef, Institute for Applied Health Research Berlin GmbH.

## Discussion

This study focused on children and adolescents with statutory health insurance as according to information from the Scientific Institute of Private Health Insurance (PKV) only 1.56 million 0- to 19-year-olds were privately insured in 2020; this proportion has a statistically small effect on the calculated prevalence and mortality values of life-threatening and life-shortening diseases in children and adolescents compared with those for children and adolescents with statutory health insurance.^5^

## Differences in the Prevalence Values of the German Association of Statutory Health Insurance Funds and Institute for Applied Health Research Berlin

The reason for the differences in the calculated prevalence values between the GKV-SV and InGef is the differences in the case definitions and groups of insured persons caused by the different health insurance funds. Accordingly, the lower prevalence value of InGef is due to the more differentiated inclusion procedure. It was not possible to align the narrower case definitions of the GKV-SV with those of InGef.

## Prevalence Values of England and Germany

The prevalence data were not directly comparable due to variations in research methodology and the use of different ICD-10 lists for calculation. However, it is important to note that the prevalence data derived from the adapted Fraser list, which identified 319 948 diagnosed cases, is far above of 50 000 used in Germany, although additional diagnoses were removed.^5^ In contrast to England, Germany differentiates between patients with statutory and PKV. There is no central register in Germany, such as NHS Digital, in which corresponding data from health and social services are documented. A comparison of the selected diagnoses with data collected in children’s hospices and specialized palliative services, as performed in the study of Fraser et al,^1^ could not be made as no corresponding data material was available.

In addition, diagnoses that do not necessarily lead to hospice and palliative care were included in the prevalence calculation because (a) they require acute (intensive) medical or nursing care, and/or (b) depending on the course of treatment, they should at least potentially be considered to require future care, even though, in the best case of successful curative therapy, they do not require permanent intensive and palliative care. These diseases are mainly found in TfSL group 1, with a small proportion subsumed under TfSL group 4; this includes cerebral hemorrhages, which can change from a life-threatening to a chronic (residual) condition.^5^

Furthermore, an increase in prevalence rates is observed in both studies, in Fraser et al’s^11^ and in the currently collected data in Germany. In England, the number of affected children and adolescents increased by 17.7% from 2014 to 2018. In Germany, during the same period, the prevalence increased by 9% to 9.2% (depending on the list). The different rates of increase in the 2 countries could possibly be related to different coding practices, accounting modalities—also country-specific—and the increase in awareness of diseases among the coding physicians. The results of this study also showed that the extrapolation of the prevalence figure from England to Germany must be reconsidered in view of the currently collected figures.

## Mortality Data in Germany

The calculation of mortality on the basis of the ICD-coded diagnoses is only of limited significance. By means of the calculations, it was possible to calculate the number of deaths from the specifically selected ICD-10 codes from 2014 to 2019, but it could not be determined to what extent these codes actually corresponded to the underlying diseases.^12^ The underlying conditions were understood as “(a) the diseases or injuries that initiated the causal chain of disease states directly leading to death, or (b) the circumstances of the accidents or violence that caused the fatal outcomes.”^13^ In the present calculations, eg, heart failure was the second most frequent diagnosis of deceased children and adolescents with life-threatening and life-shortening diseases, although it is a secondary disease that precedes another underlying condition such as coronary heart disease.^12^

## Conclusion

It should be noted that the results do not provide direct conclusions regarding the quantitative care needs of the target group. Additional data on the needs of children, adolescents, and their families in various phases of illness^14^ are required. In addition, the diversity of disease courses and the resulting needs for medical and nursing care as well as psychosocial support and counseling must also be taken into account in the comparison of care. When considering both prevalence and mortality values, a mortality rate of 1458 children and adolescents with life-threatening and life-shortening diseases indicates that a relatively small proportion of patients with these conditions pass away compared with their prevalence. Although the data cannot directly determine the care needs, it can be assumed that extended care phases should be incorporated into the structures of accredited health care providers and clinics, as well as in child and youth hospice care. The prolonged illness phases and the relatively high life expectancy derived from the data should also be considered when designing effective transition processes and structures from adolescent to adult care settings. To provide more conclusive statements in this regard, further investigations are necessary, including studies that address the recording of care structure data, the exemplary collection of case studies of affected children and adolescents, and the analysis of potential care needs on the basis of subgroups of the TfSL diagnosis groups.

## Limitations

The prevalence range determined in Germany was based on accounting data of documented inpatient and outpatient diagnoses, which have limitations in their clinical verifiability and validity.^15^ The selection of ICD-10 codes and their categorization into life-threatening and life-shortening diseases, as well as their assignment to TfSL groups, could vary from a professional standpoint, despite maximum dialogue validity achieved through discussions among collaborating physicians. Another limitation arises from the classification system of the ICD-10, which does not allow for more differentiated categorization of symptoms, syndromes, and clinical conditions, often requiring them to be grouped together.^16^ For example, the code E74 (Other disorders of carbohydrate metabolism) was included in the prevalence calculation, after consultation with physicians, as hereditary galactose or fructose metabolism disorders can lead to life-threatening crises in newborns.^17^ However, the ICD-10 coding system does not provide a clear distinction for these diagnoses, such as non–life-threatening intestinal fructose intolerance. The lack of differentiation is a consequence of the structure and limited granularity of the ICD-10.^5^ Furthermore, in the German health care system, issues related to diagnosis-related group (DRG) gaming and upcoding can impact billing practices, leading to potential overdocumentation and the main discharge diagnosis not accurately reflecting the underlying reason for the hospital stay.^16^

Project PraeKids implemented feedback loops with experts to ensure high content validity. Medical professionals conducted random checks of classifications in the TfSL groups after group discussions and achieved agreement. In addition, an experienced health services researcher reviewed the results. However, due to limitations, a validity check of internal and external validation through sample comparisons was not feasible.^16^

When classifying the TfSL groups, it is crucial to consider that individual diagnoses may change over time. For example, a life-threatening condition initially (TfSL-1), such as a grade 3 brain hemorrhage, could evolve into a chronic residual condition (TfSL-4). Furthermore, advancements in medical science, such as innovative surgical procedures and novel medications, are providing additional perspectives in disease treatment, potentially transforming previously life-shortening conditions into manageable or even curable ones over time. Consequently, the selected ICD codes for the diseases included in the study must be dynamically assessed and possibly adjusted for studies. It is also to be considered that the results were scaled to the population size without accounting for other sociodemographic distribution characteristics as the data from GKV-SV and InGef lacked information on patients’ living conditions.

Furthermore, no statements can be made about the uncertainties or decision criteria used by physicians in clinical diagnosis and documentation as the survey was conducted using secondary data, whereby a certain variance in outpatient and inpatient diagnoses can be assumed.^18,19^ For billing purposes, a principal diagnosis and optional secondary diagnoses are determined at discharge.^20^ It can therefore be assumed that there is a corresponding variance in diagnoses, which can be shown, eg, in different admission and discharge codes for a child. The resulting code changes could not be taken into account in the study as only the discharge diagnosis code or the last diagnosis code from the billing quarter was included.

With regard to mortality, it should be noted that underlying causes of death are not uniformly recorded and processed in practice.^21,22^ Accordingly, they may be subject to errors. Wengler et al point out that inaccuracies can arise in the digital processing of information from death certificates for the purpose of statistical processing or in the determination of the cause of death by medical professionals. This is due to the lack of generally valid and explicit regulations on what can be considered a cause of death and which causes can be excluded.^12^

## Author Contributions

NMB and SJ conceptualized and designed the study; analyzed and interpreted the data; drafted the initial manuscript; reviewed and revised the manuscript; approved the final manuscript as submitted.
